# Synthesis, Tribological and Hydrolysis Stability Study of Novel Benzotriazole Borate Derivative

**DOI:** 10.1371/journal.pone.0083501

**Published:** 2014-01-22

**Authors:** Xiong Liping, He Zhongyi, Qian Liang, Mu Lin, Chen Aixi, Han Sheng, Qiu Jianwei, Fu Xisheng

**Affiliations:** 1 School of Basic Science, East China Jiaotong University, Nanchang, Jiangxi, China; 2 Shanghai Institute of Technology, Shanghai, Shanghai, China; 3 PetroChina Lanzhou Lubricating Oil R&D Institute, Lanzhou, China; University of Akron, United States of America

## Abstract

Benzotriazole and borate derivatives have long been used as multifunctional additives to lubricants. A novel, environmentally friendly additive borate ester (NHB), which contains boron, ethanolamine, and benzotriazole groups in one molecule, was synthesized by a multi-step reaction, and its tribological properties in rapeseed oil (RSO) were investigated by a four-ball tribometer. The hydrolysis stability of the additive was investigated by half-time and open observation methods, and the mechanism of hydrolysis stability was discussed through Gaussian calculation. The novel compound NHB showed excellent performance under extreme pressure, against wearing, and in reducing friction, and its hydrolysis time is more than 1,220 times, which is better than that of triethyl borate. The mass ratio of NHB is bigger than that of the mixed liquid of triethyl borate and ethanolamine. The lone electron of amino N atoms forms a coordination effect with the B atom to compensate for the shortage of electrons in the B atom and to improve the hydrolysis stability of NHB. The surface morphology and the traces of different elements in the tribofilms formed with 1.0 wt.% NHB in were detected with scanning electron microscopy(SEM), energy dispersive X-ray spectroscopy (EDX)and X-ray photoelectron spectroscopy(XPS). The results shown that the additive caused a tribochemical reaction with the steel ball surface during the lubricating process. A mixed boundary lubrication film that contains organic nitrogen and inorganic salts, such as BN, B_2_O_3_, FeOx, Fe–O–B, and FeB, was also formed, and the formation of the lubricating film improved the tribological properties of the base oil.

## Introduction

Pure base stock cannot meet all of the stringent requirements of modern industry. For this reason, some functional additives must be added to suit the working condition. Traditional lubricant additives face enormous challenges with the growing requirements of environmental protection for lubricating oil [1]. Some organic derivatives that contain tribologically active elements (P, S, N, Cl, Zn, Mo, and B) are admixed to oils as additives at relatively low concentrations [2]. The main tribochemical reactions will occur during contact among surface materials, base stock, and lubricating oil additives in the boundary. The presence of reactive metals, which are exposed by the wear process, can both catalyze lubricant breakdown and initiate chemical interactions with the molecules of the lubricant additives [3].

Boron-based lubricant additives have recently received significant attention because of their wear-reducing and frictional properties as well as low pollution [4]. Some studies showed that borate ester possesses robust extreme pressure and antiwear properties, especially in low viscosity oil [5].

Some simple boron compounds, such as borate salt or borate ester, have also been used as corrosion inhibitor, antioxidant, friction modifier, as well as extreme pressure and antiwear additive in environment-friendly base stock. These organic borate ester compounds are directly dissolved in lubricating oils, but inorganic borate salts, such as OLOA 9750 (potassium borate), are dispersed as insoluble nanoparticles [6]. The antiwear mechanism of B compounds in extreme pressure conditions is due to the formation of thin layers of boric oxide (B_2_O_3_) on the metal surfaces [7]. Boric oxide is converted to boric acid (H_3_BO_3_) upon exposure to humid air, which is a layered material with a specific structure, whereby the atoms are covalently bonded to each other, and the layers are weakly bonded. When the layers are stressed, they shear and slide over one another easily, providing low friction [8]. Some N-containing heterocyclic compounds, such as benzotriazole and triazine, have been used as lubricating oil additive. These compounds possess viable tribological performances. Li Jiusheng et al. [9] designed and synthesized a novel B derivative of benzotriazole by combining N, B, and O atoms in one individual compound. Their study showed that both benzotriazole and alkyl borate groups may simultaneously react on steel surfaces, and these reactions may be activated by shear and/or thermal effects at surface asperities, which explains the superior tribological properties of the additive.

However, the low hydrolysis stability of borate salts and borate esters is the biggest limitation for industrial application. Stability is easily attacked by water due to the lack electrons of the B atom. The attack will cause the loss of the effective additive B component, which decreases the tribological performances of the lubricating oil [10] and limits its practical application. Some studies showed that the addition of nitrogen-containing materials, such as amine, will improve the hydrolysis stability of borate salts and esters. N-containing heterocyclic compounds are reported to possess excellent extreme pressure and antiwear properties in lubricating oil. The number of N atoms is the main factor that affects tribological performance. The N-containing heterocyclic compound contains N atoms, which possesses a lone electron pair in the *p* orbital that will form a complex with the empty 2*p* orbital of the B atom to reduce the possibility of attack by some nucleophiles, such as water.

The ethanolamine and benzotriazole ethanol groups, which increase the number of N atoms in the borate ester molecule, are introduced in the synthesis of a novel borate ester (NHB) in the present work. The effects of both benzotriazole substitute and ethanolamine group at the alkyl borate part of the molecule on the tribological performance of rapeseed oil (RSO) are investigated in a four-ball machine. Finally, both roughness and the elemental composition of wear scars on the steel balls after the tribological tests are analyzed using scanning electron microscopy/energy dispersive X-ray spectroscopy (SEM/EDS) and X-ray photoelectron spectroscopy (XPS). The hydrolysis stability was tested using the open method and half-time method, and the Gaussian calculation was used to investigate the hydrolysis mechanism.

## Additive Synthesis and Analysis Methods

### 2.1 Additive synthesis

**Figure 1:**
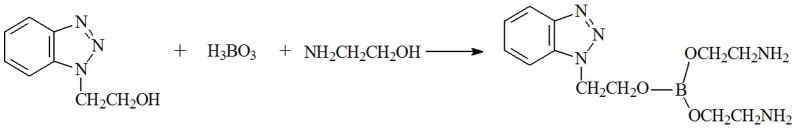
Synthesis route of NHB.

The analytical reagents boric acid and ethanolamine were obtained from Shanghai Shenbo Chemical Industry Co., Ltd. Benzotriazole ethanol was synthesized in the laboratory, whereas trimethyl borate, triethyl borate, and tributyl borate were industrial products. Figure 1 shows the synthesis route of borate ester.

Benzotriazole ethanol (0.1 mol), boric acid (0.1 mol), and ethanol amine (0.2 mol) were added to a four-mouth flask containing toluene as solvent and water-carrying agent; the mixture was then stirred and heated. The reaction ended when the generated water in the water separator was equal to the theoretical yield. Then, vacuum distillation was performed, and a yellow viscous liquid benzotriazole borate derivative (NHB) was obtained. The production of NHB is about 26.8031 g.The structure of the product was analyzed by infrared (IR) and elemental analyses. The elemental analysis results were as follows (data in parentheses are calculated values): C%, 48.60(49.17); N%, 23.71 (23.89); H%,6.92(6.88); and B%,3.77(3.69).

The base oil, natural RSO, used in the present study was produced by Jiujiang Co., Ltd. (Jiangxi Province). Its physical and chemical properties are as follows: 0.80 mg KOH/g; viscosity index, 214; ν40°C, 38.60; and basic N concentration, 0.1975 mg/g.

### 2.2 Analysis methods

#### 2.2.1 Specimens and testing apparatus

All the steel balls (φ 12.7 mm) used in the test were made of GCr15 bearing steel, with hardness of HRc 59 to HRc 61. A microscope was used to determine the wear scar diameter (WSD) of the three lower balls with an accuracy ±0.01 mm.

The Chinese standard test method GB 3142-82, similar with ASTM D2783, was used to evaluate the maximum non-seizure load at a rotation speed of 1,450 rpm for 10 s at room temperature. Friction and wear tests were examined on a four-ball test machine made in Jinan Testing Machine Factory of China at a rotation speed of 1,450 rpm at different loads for 30 min.

#### 2.2.2 Hydrolysis stability

Hydrolysis stability was investigated by an open method. Borate ester with 10 ml 1.0 wt.% concentration in liquid paraffin was added to a 25 ml dry glass, and changes in turbidity were observed. The turbidity of the liquid indicates the generation of insoluble boric acid solid. The time spent for turbidity change was used as the hydrolysis stability time.

Hydrolysis stability was investigated by another method, namely, the half-life method. Approximately 50 ml of water and 5 ml of glycerin were mixed in a glass, added with 3 to 4 drops of phenolphthalein solution and 0.1 mol/l NaOH solution and then mixed again. This mixture was then added into a beaker with 0.4–1.2 mmol borate ester and twice the amount of NaOH. The solution was stirred continuously until the color changed from red to colorless. The time spent for the change to occur was considered the hydrolysis stability time.

#### 2.2.3 Computing method

All the calculations were completed at the Nanchang University computing workstation. The geometrical configuration of borate ester in the hydrolysis process was optimized with a program of Gaussian 03 density functional method, in B3LYP/6-31G* level. The charge quantity and atom distance of borate ester molecules were calculated to obtain the reasonable space structure [11].

#### 2.2.4 Worn surface analysis

WSD was analyzed with a PHI-5702 type XPS. The radiation source was Mg K_α_ with pass energy of 29.35 ev, and the binding energy of C_1s_ (284.6 ev) was used as a standard value. The SIGMA type field emission SEM (Zeiss Company) was used to study the rubbed surface morphology.

## Results and Discussion

### 3.1 P_B_ value

The P_B_ values of the RSO containing different concentrations of NHB are shown in Table 1. The table shows that the addition of NHB in RSO improved the extreme pressure properties of the RSO to a large degree. It can improve P_B_ value over 190% than that of base oil when NHB concentration is 0.7 wt %, this improvement indicated that NHB possesses good extreme pressure property as additive in lubricating oil. The P_B_ values increased when the NHB concentration was less than 1.0 wt.%, but did not increase at concentrations higher than 1.0 wt.%.

**Table 1:** P_B_ values of oil samples.

Oil sample	RSO	0.1 wt.%	0.3 wt.%	0.5 wt.%	0.7 wt.%	1.0 wt.%	2.0 wt.%
P_B_ value (N)	696	921	980	1185	1323	1254	1048

### 3.2 Antiwear performance

**Figure 2:**
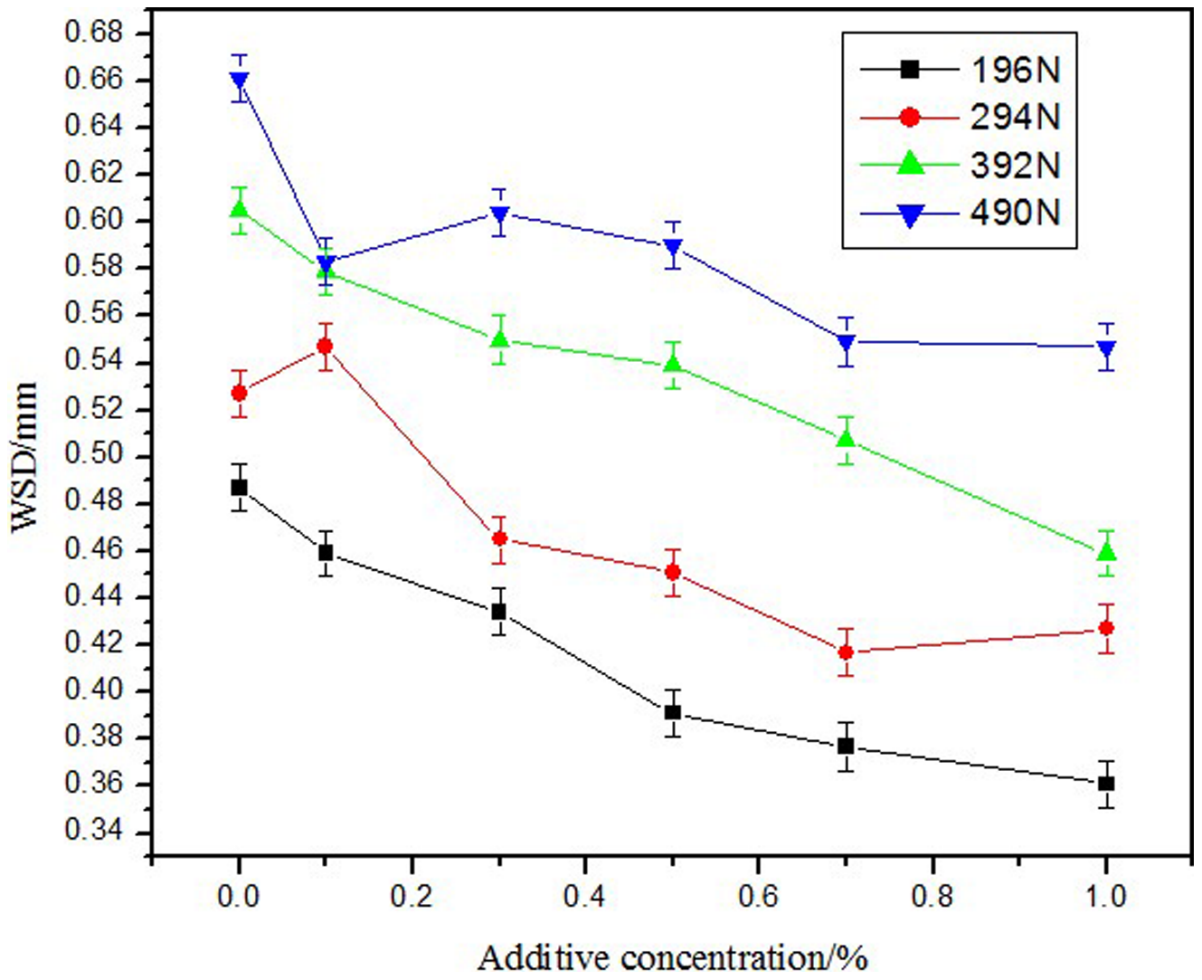
Effect of additive concentration on WSD.

Figure 2 shows the WSD of RSO at an applied load of 196, 294, 392, and 490 N in different NHB concentrations. RSO showed robust antiwear behaviors upon the addition of NHB in base oil at different applied loads. The increase in the mass concentration of NHB in the base oil and the decrease of WSD indicated improved antiwear capabilities.

**Figure 3:**
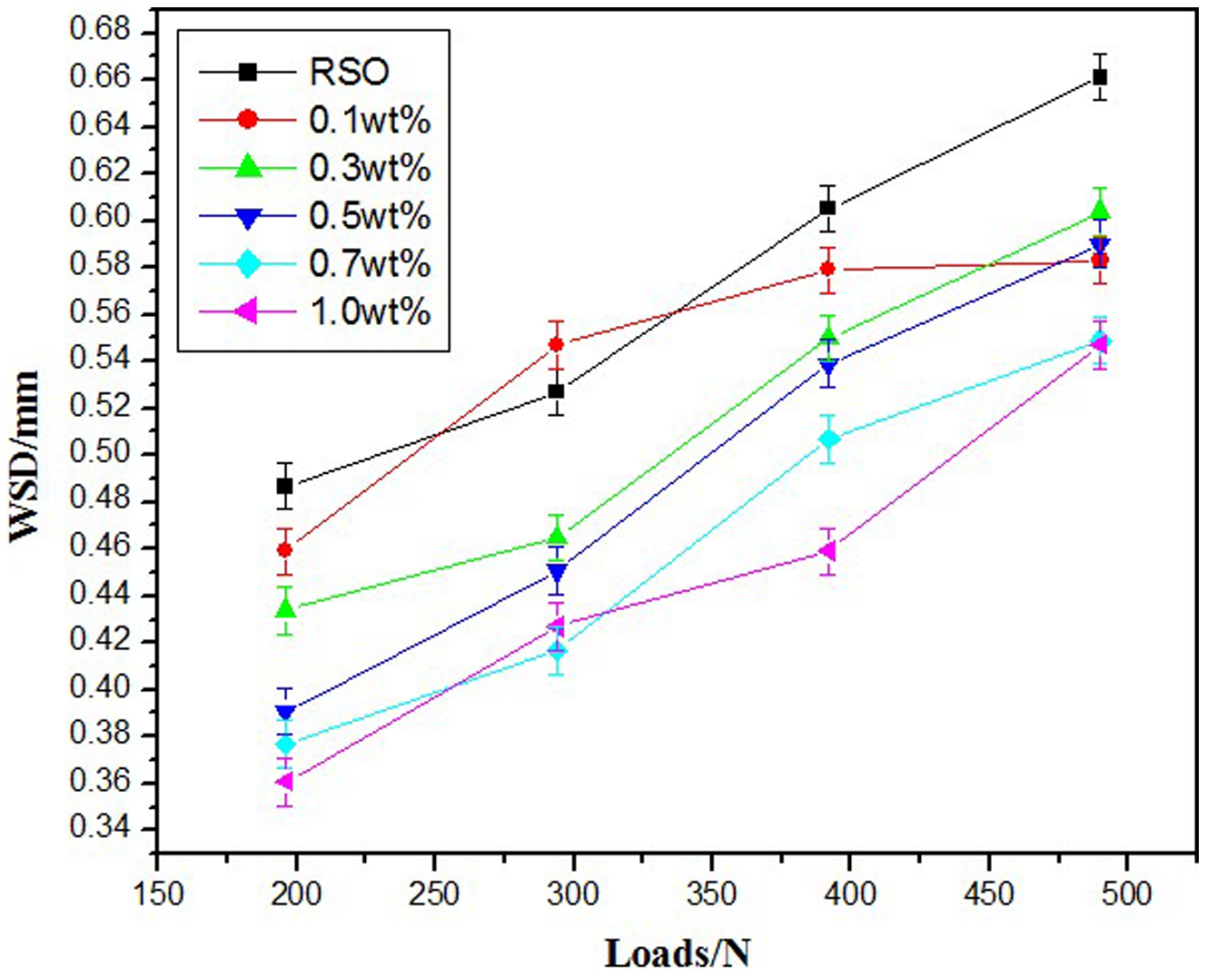
Effect of applied load on WSD.

Figure 3 shows the WSD at different concentrations of NHB in RSO at different applied loads. The WSD increased with the increase of the applied load. The WSD of the samples containing additive oil was far less than that of the blank RSO. Higher additive concentration resulted in lower WSD and better antiwear performance.

### 3.3 Friction-reducing performance

**Figure 4:**
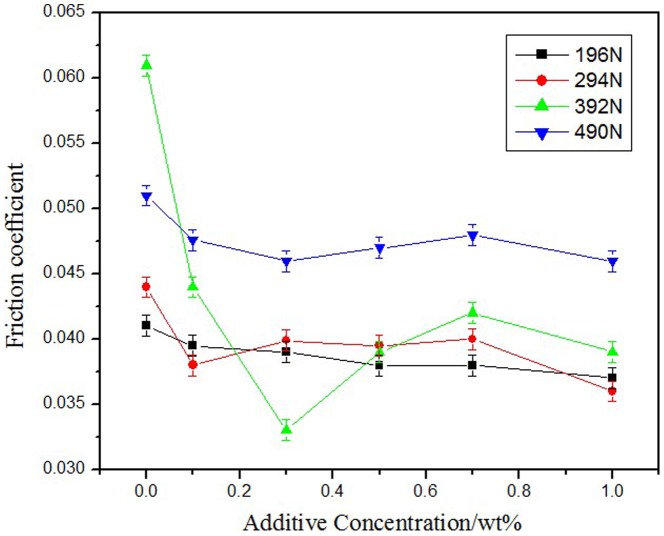
Effect of NHB concentration on friction coefficient.

Figure 4 shows the relationship between the friction coefficient and the different additive concentrations at 196, 294, 392, and 490 N. The synthesized additive NHB could reduce the friction coefficient at the range of the applied load. The friction-reducing effect became smoother when the additive mass concentration was more than 0.3 wt.%, and the friction coefficient of the base oil was reduced close to 50% at 0.3 wt.% concentration at 392 N.

**Figure 5:**
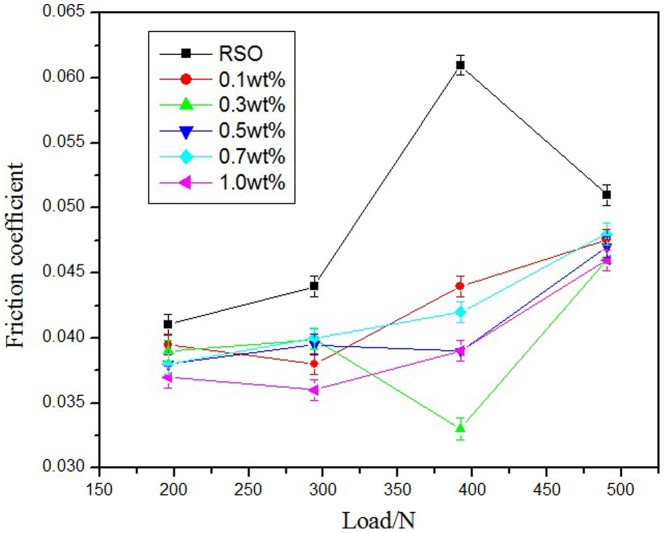
Effect of load on friction coefficient.

Figure 5 shows the relationship between the friction coefficient and the applied load at different additive concentrations. The different concentrations of the synthesized additive RSO all reduced the friction coefficient in the applied load test. The friction coefficient values were lower than those of the base oil at the applied load from 196 N to 490 N, and the lubricating oil at 1.0 wt.% concentration possessed better friction-reducing property at any applied load.

### 3.4 Hydrolysis stability

The turbidity of petroleum wax oil did not change after two months at room temperature when 1.0 wt.% NHB was added. The turbidity time of trimethyl borate and tributhyl borate was 5 min and 10 min, respectively. These findings indicated that NHB possessed excellent hydrolysis stability due to the presence of benzotriazole and ethanolamine, which contain the N element. The lone pair electrons of the N atom made up for the electron-deficiency of the B atom.

Borate ester was added with 0.2 g water to observe the turbidity time of liquid in a constant temperature of 70°C and to shorten the turbidity time. The change in time was marked as the hydrolysis time. The components and results are shown in Table 2. The hydrolysis time of NHB was 76,980 s, which was 1,220 times that of triethyl borate, and also bigger than that of the mixture of different concentrations of ethanolamine and triethyl borate. Thus, to make up for the B electron deficiency of ethanolamine, the inner coordination with borate was better than that of the outer coordination with triethyl borate.

**Table 2:** Hydrolysis time of the different samples.

Samples	Paroline (g)	Triethyl borate(g)	H_2_O(g)	NH_2_CH_2_CH_2_OH (g)	Hydrolysis time(s)	Deviation of Hydrolysis time(s)
Black	24.80	0.25	0.20	0.00	63	±1
A	24.80	0.25	0.20	0.25	233	±1
B	24.80	0.25	0.20	0.50	307	±1
C	24.80	0.25	0.20	0.75	344	±1
D	24.80	0.25	0.20	1.00	410	±1
E	24.80	0.25	0.20	1.25	463	±1
NHB	24.80	0.25	0.20	0.00	76980	±6

The root cause of borate ester hydrolysis results is the *sp2* hybridization of the B atom [12]. An empty 2*p* orbital exists and is easily attacked by the nucleophile, which has a lone electron pair. The attack can increase the bonding action between the empty 2*p* orbital and lone electron pair. On the other hand, the water molecule contains a lone electron pair that can attack the B atom in the borate ester and then contribute to its hydrolization. According to extant research, the hydrolysis process of borate ester is accomplished in three steps: first, the borate ester is attacked by water, then, the unstable tetrahedral complex is generated, and finally, alcohol is desquamated.

### 3.5 Worn surface analysis

**Figure 6:**
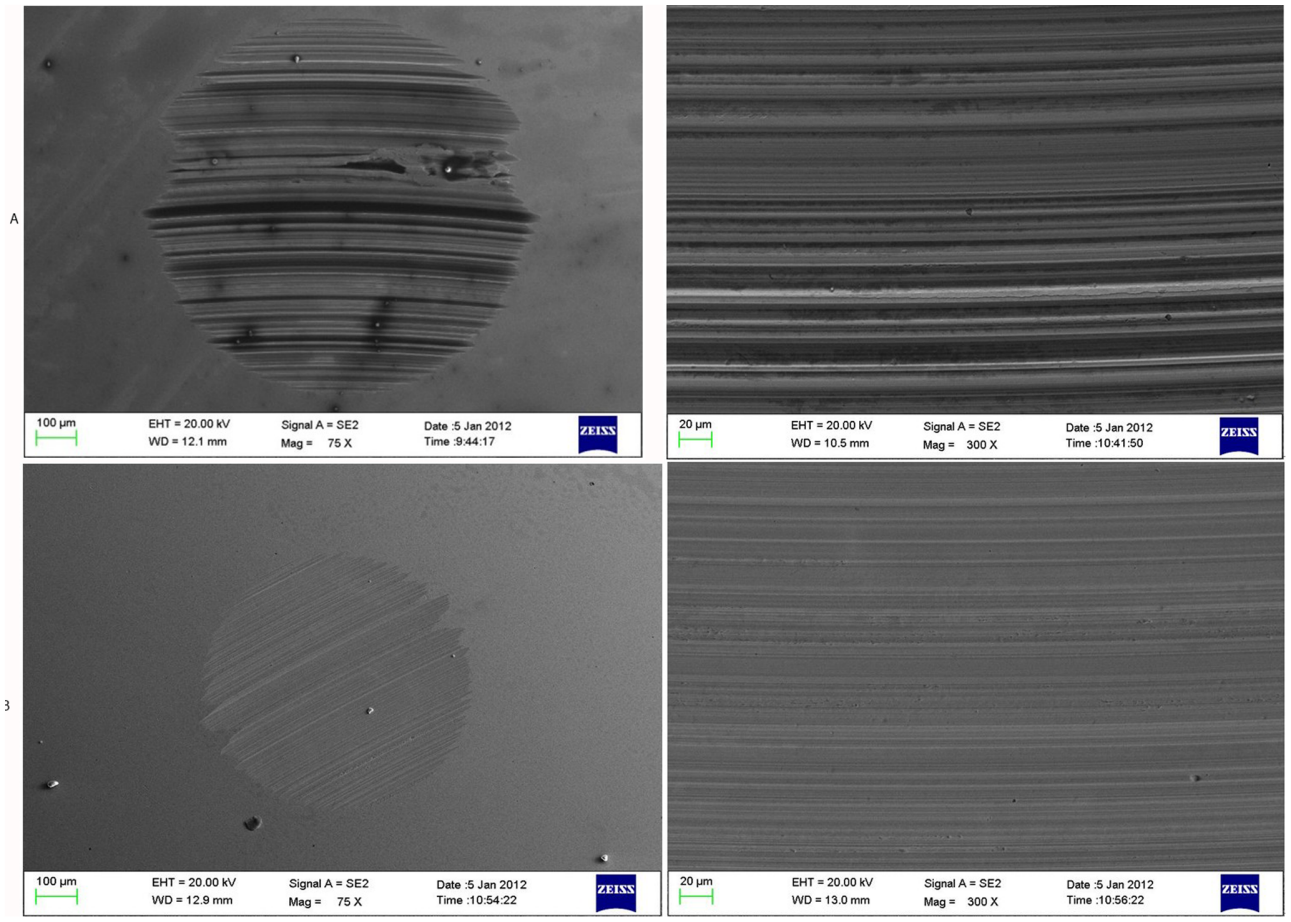
SEM photos of the surface of steel ball. (a) RSO. (b) RSO containing 1.0 wt.% NHB.

To understand the boundary lubricating mechanism of the additive in the lubricating process better, this work used SEM to study the worn surface of the steel ball. Figure 6 shows the SEM results of the worn surface of the steel balls lubricated by RSO and RSO with 1.0 wt.% NHB under the applied load of 392 N.

The SEM micrographs in the figure indicate that severe scuffing occurred with the lubrication of base oil alone based on the grain abrasion characteristics [13]. The lubrication of RSO containing 1.0 wt.% synthesized additive NHB showed only slight frictional tracks with no obvious deep furrows. This observation indicated that NHB can improve the tribological properties of base oil. The N and B active elements in the additive NHB [14] effectively formed a layer of B nitride films to cover the surface of the friction pair during the lubricating process, which thereby reduced friction.

**Figure 7:**
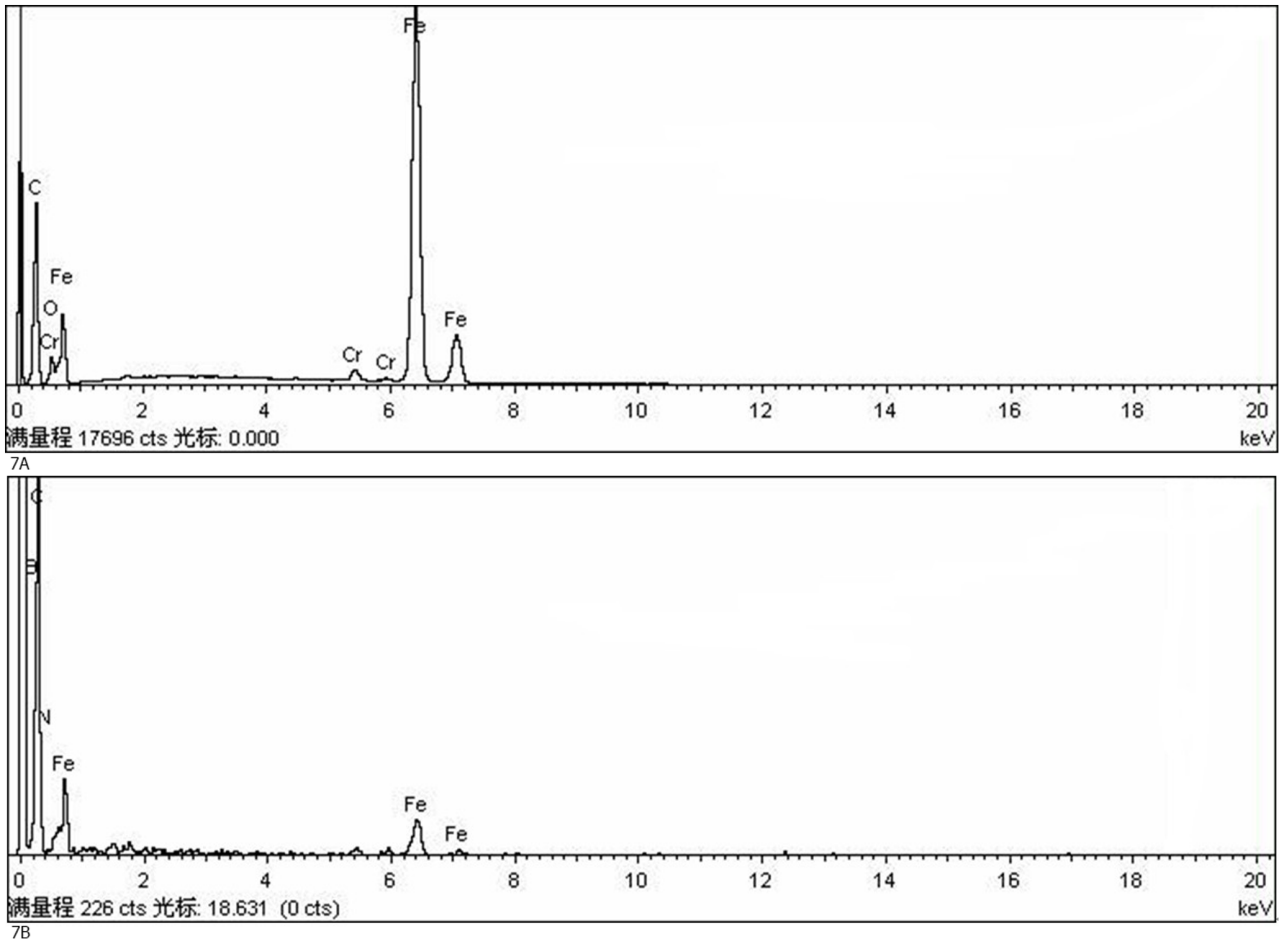
EDS result of the surface of steel ball. (a)Base oil. (b) Base oil containing 1.0 wt.% NHB.

Figure 7 shows the steel ball surface elements content and distribution analysis of EDS, which offers a more intuitive analysis of tribochemical reactions on the steel ball surface during the lubricating process. The elements on the steel ball surface were Fe, Cr, and C when lubricated with RSO only as the material. The worn surface of the steel ball showed B and N elements, which only came from additive NHB when using 1.0 wt.% NHB as additives in RSO. Fe and Cr element concentrations were lower than that of the base oil during the lubricating process.

Table 3 shows the different elements on the worn surface. When the worn surface had N, B elements, the antiwear properties increased. This increase was because when NHB was introduced to the base oil, the N, B active elements of NHB effectively combined to form boron nitride film [9], [15], covering the steel ball worn surface to stop further wear.

**Table 3:** Elements on the surface of the steel balls.

Element	C	B	N	Fe	Cr
Basic oil	71.34	0	0	22.64	0.39
1.0 wt.% NHB	72.07	22.85	3.92	0.96	0.20

### 3.6 XPS analysis results

**Figure 8:**
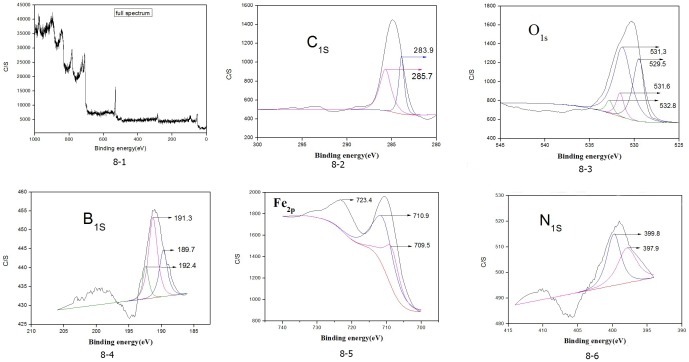
XPS spectra of the characteristic elements of NHB on the worn surface.

The worn surfaces of the steel/steel pairs lubricated with the RSO containing 1.0 wt.% NHB under 392 N were analyzed by XPS. The analysis obtained more information on the tribochemical reactions involved during the sliding process. Figure 8 shows the results of the analysis.

The binding energies of C_1s_ were 285.7 and 283.9 eV, which corresponded to the C–H, CO–, COO– in the additive and base oil. Thus, the base oil and additive were adsorbed on the metal surface. The binding energies of Fe_2p_ were 710.9, 709.5, and 723.4 eV, which corresponded to iron oxides [6], which were also supported by the binding energies of O_1s_ at around 533 eV. The binding energies of O_1s_ were 529.5, 531.6, and 532.8 eV, which corresponded to Fe_2_O_3_, Fe–O–B, and FeO. The peak in 531.3 eV of O_1s_ corresponded to the COO– of the base oil. The broad peak of B_1s_ from 191.0 to 192.4 eV was attributed to BO_x_ and O–Fe–B, among others [16]. These data indicated that the formation of the B element was adsorbed on the surface with boron oxygen, which suggests the presence of degraded borate ester on the top surface. The binding energy of B_1s_ at 189.7 eV corresponded to the BN form, which suggests that the presence of a small amount of degraded borate esters that reacted with steel produced the BN compound. As for the N element on the surface, the binding energy of N_1s_ was located at 399.5 eV, which can be assigned to the adsorption of the organic N-containing compounds on the overlayer [17]. The N_1s_ spectrum showed two peaks at 399.9 and 398.7 eV, indicated the existence of inorganic N-containing compounds. The binding energy of N_1s_ were 399.8 and 397.9 eV, which corresponded to organic N [18] and BN, respectively. Moreover, these compounds were also supported by the binding energies of B_1s_ at around 189.7 eV. Hence, the N element of amine was adsorbed on the steel ball surface with organic amine and BN, which were produced during the tribological process whereby the NHB had a tribochemical reaction with the steel ball surface.

Figure 8 XPS spectra of the characteristic elements of NHB on the worn surface

In conclusion, the tribofilm containing N, B, O, and Fe elements were formed on the worn surfaces under the lubrication of the RSO that contained the NHB during the sliding process, as confirmed through XPS spectra analysis. The resulting surface protective films contributed to the significant reduction in friction and wear.

According to the above results, the tribochemical mechanism of NHB as additives in RSO is discussed below.

First, the additive was adsorbed on the metal surface, taking on competitive adsorption with RSO in the lubricating process [19]. The tribological reaction between metals produced a partially high temperature that resulted in the tribochemical reaction of the additive with the steel ball surface. The additive molecule decomposed, and the ethanolamine group reacted with the metal surface to form organic N and N-containing metal complex film. Subsequently, the benzotriazole ethanol group was adsorbed on the surface. The borate group reacted with the metal surface to form inorganic boron oxygen film, which possessed a high degree of hardness, contributing to the higher carrying capacity of the tribological surface film. The inorganic and organic protective film in the metal surface had a certain tribological performance.

Therefore, a mixed reaction boundary film existed in the worn steel surfaces caused by the chemical composition, base oil, and additive in the lubrication process. The lubricant film formation improved the tribological properties of the base oil.

In short, the tribological mechanism of the synthesized borate ester derivative showed that the additive underwent a chemical reaction with the steel ball surface and then formed a complex boundary lubricating film that contained organic N-containing compounds and inorganic salts, such as FeB, Box, and BN, among others. The formation of lubricating films improved the tribological properties of the base oil.

## Hydrolysis Stability Mechanism

**Figure 9:**
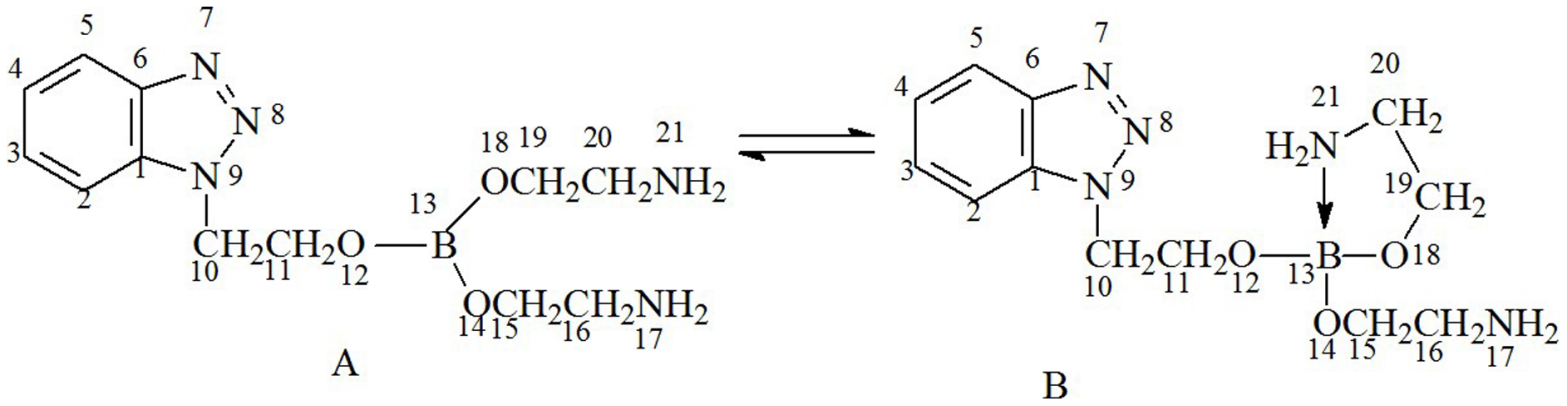
Space structure and atom label of NHB.

The space structure of benzotriazole ethanol borate ester was evaluated by Gaussian software Gauss03 Revision-E.01. The space structure and atom label of NHB are shown in Figure 9. Structure A had no optimization, whereas structure B was optimized. The figure shows that N atoms in structure A undergo planar movement, and in structure B, the N atom (labeled 21) moves toward B atom (labeled 13). Thus, the N and B atoms may form a coordinated bond that allows electrons to move from the N atom to the B atom.

### 4.1 Analysis of atom charges

Table 4 shows the atom charges. When structure A was transformed into structure B, the charge of N atoms (labeled 17 and 21) decreased, whereas the charge of N atoms (labeled 7, 8, and 9) did not change significantly with their move from structure A to structure B. Hence, the charge of N atoms in ethanolamine flowed toward the B atom, but the charge of the N atoms in benzotriazole did not flow toward the B atom.

**Table 4:** Charges of the different atoms.

	B (13)	N (7)	N (8)	N (9)	N (17)	N (21)
A	0.563215	−0.362562	−0.050341	−0.404368	−0.706369	−0.722865
B	0.555046	−0.363324	−0.050256	−0.404153	−0.115428	−0.108396
Trimethyl borate	0.781644					
Triethyl borate	0.780481					
Tributyl borate	0.766041					
Benzotriazole		−0.286358	0.023389	−0.313312		
Benzotriazole ethanol		−0.363809	−0.050869	−0.398060		

### 4.2 Separation distance between the B atom and different N atoms

Table 5 shows the separation distance between the B atom and different N atoms. The Table 5 shows that with the optimization of the molecule, the N atom in amino moved closer toward the center B atom, but the N atoms in benzotriazole were far away from the center B atom.

**Table 5:** Separation distance between the B atom and different N atoms.

Atom	13–21	13–17	13–7	13–8	13–9	12–13
A (non-optimized) Å	4.86500	4.94673	6.77377	5.70774	4.90077	1.381
B (optimized) Å	3.49692	4.89883	6.79369	5.71773	4.91206	1.3755

From the above results, the Gaussian calculation showed that the hydrolysis stability of NHB may be attributed to the lone electron pair of amino N atoms that form a coordination effect with the B atom to compensate for the shortage of electrons of the B atom, as seen from the charge of the B and N atoms as well as the distance between the N atoms and B atom.

## Conclusions

The following conclusions can be drawn from above results:

1. The antiwear and extreme pressure properties of NHB increased with the increase of the additive concentration. P_B_ values increased with the increase of the NHB concentration under 1.0 wt.%, but was not increase at concentrations higher than 1.0 wt.%. The antifriction effect becomes smoother when the additive mass concentration is more than 0.3 wt.%, and the reduction of the friction coefficient of the base oil is close to 50% at 0.3 wt.% NHB at 392 N.
2. The addition of 1.0 wt.% NHB in petroleum wax oil did not change the turbidity, even after two months. The turbidity time of trimethyl borate and tributhyl borate are 5 min and 10 min, respectively. The hydrolysis time of NHB is more than 1,220 times than that of triethyl borate and is more than that of the mixed liquid of triethyl borate and ethanolamine with any mass ratio. The lone electron of amino N atoms was formed a coordination effect with the B atom to compensate for the shortage of electrons in the B atom and improved the hydrolysis stability of NHB.
3. The NHB has a tribochemical reaction during the sliding process, and the N element of amine is adsorbed in the steel ball surface with organic amine and BN, whereas other groups form inorganic salts. XPS spectra and EDS analysis show that the complex tribofilms containing B, N, O, and Fe elements are formed on the worn surfaces under the lubrication of the RSO containing NHB. The resulting surface protective films contribute to reduce the friction and wear significantly.

## Author Contributions

Conceived and designed the experiments: XL. Performed the experiments: HZ QL.Analyzed the data: QJ FX. Contributed reagents/materials/analysis tools: CA HS. Wrote the manuscrip: HZ ML.
